# Current Management of Post-radical Prostatectomy Urinary Incontinence

**DOI:** 10.3389/fsurg.2021.647656

**Published:** 2021-04-09

**Authors:** Mohammad S. Rahnama'i, Tom Marcelissen, Bogdan Geavlete, Manuela Tutolo, Tanja Hüsch

**Affiliations:** ^1^Urology Department, Uniklinik RWTH Aachen, Aachen, Germany; ^2^Maastricht University Medical Center (MUMC+), Maastricht, Netherlands; ^3^Bucharest University Emergency Hospital, Bucharest, Romania; ^4^Division of Oncology, Unit of Urology, Urological Research Institute, IRCCS Ospedale San Raffaele, Milan, Italy; ^5^Mainz University Hospital, Mainz, Germany

**Keywords:** prostate cancer, incontinence (male), detrusor activity, stress incontinence, prostatectomy

## Abstract

Prostate cancer is the second most common cancer in men worldwide. Radical prostatectomy and radiation beam therapy are the most common treatment options for localized prostate cancer and have different associated complications. The etiology of post prostatectomy incontinence is multifactorial. There is evidence in the literature that anatomic support and pelvic innervation are important factors in the etiology of post-prostatectomy incontinence. Among the many surgical and technical factors proposed in the literature, extensive dissection during surgery, damage to the neurovascular bundle and the development of postoperative fibrosis have a substantial negative impact on the continence status of men undergoing RP. Sparing of the bladder neck and anterior, and possibly posterior, fixation of the bladder-urethra anastomosis are associated with better continence rates. Overactive bladder syndrome (OAB) is multifactorial and the exact role of prostate surgery in the development of OAB is still under debate. There are several variables that could contribute to detrusor overactivity. Detrusor overactivity in patients after radical prostatectomy has been mainly attributed to a partial denervation of the bladder during surgery. However, together with bladder denervation, other hypotheses, such as the urethrovesical mechanism, have been described. Although there is conflicting evidence regarding the importance of conservative treatment after post-prostatectomy urinary incontinence, pelvic floor muscle training (PFMT) is still considered as the first treatment choice. Duloxetin, either alone or in combination with PFMT, may hasten recovery of urinary incontinence but is often associated with severe gastrointestinal and central nervous side effects. However, neither PFMT nor duloxetine may cure male stress urinary incontinence. The therapeutic decision and the chosen treatment option must be individualized for each patient according to clinical and social factors. During the recent years, the development of new therapeutic choices such as male sling techniques provided a more acceptable management pathway for less severe forms of urinary incontinence related to radical prostatectomy. Following this perspective, technological improvements and the emergence of new dedicated devices currently create the premises for a continuously positive evolution of clinical outcomes in this particular category of patients.

## Introduction

Prostate cancer is the second most common cancer in men worldwide, affecting ~1.1 million men per year (1). Radical prostatectomy (rPR) and radiation beam therapy are comparable treatment options for localized prostate cancer (2) whereas treatment associated complications and incidences differ significantly.

Male stress urinary incontinence (SUI) has a predominantly iatrogenic cause after radical prostatectomy (3). It is defined by the complaint of involuntary leakage on effort or exertion or on sneezing, or coughing (4, 5). The mechanism for post-radical prostatectomy incontinence remains unclear (6), however, several hypothesis have been discussed. Despite direct injury to the internal sphincter itself, injury to the external rhabdosphincter or its shortened lengthwise (7), injuries to the supporting structures of the urethra (7), lesions to the nerve supply (6) or even detrusor underactivity (8) may impair continence.

The incidence of post-radical prostatectomy incontinence has become an increasingly common urological problem with a prevalence of 2.5–90% (9) depending on the definition for urinary continence. In a recent prospective non-randomized trial comparing open retropubic rPR and robotic assisted rPR including a total of 2,625 men, urinary incontinence defined by no change pad in 24 h after 12 month follow-up was 21.3 and 20.2% for robotic-assisted and open rPR, respectively (10). A meta-analysis did not identify a significant difference of urinary continence in comparison between open retropubic and robot assisted rPR (11, 12). A prospective randomized trial comparing laparoscopic and robotic-assisted rPR demonstrated significant better continence rates for robotic-assisted than laparoscopic rPR (95.0 vs. 83.3%) (13). A meta-analysis identified evidence for improved continence rates with robotic-assisted in comparison to laparoscopic rPR accordingly (14). Table 1 present the continence rates after radical prostatectomy reported by selected prospective trials.

**Table 1:** Continence rates after radical prostatectomy of selected clinical trials.

**References**	**Year**	**Study design**	**Number of patients**	**Follow-up time**	**Continence definition**	**Urinary continence, n/N (%)**
Haglind et al. (10)	2015	Prospective, non-randomized	2,625	12 months	<1 pad/24 h	RALP 366/1847 (21.3) RRP 144/778 (20, 2)
Choo et al. (15)	2012	Prospective, non-randomized	253	24 months	0–1 pad/24 h	RALP 73/77 (95) RRP 172/176 (98)
Rocco et al. (16)	2009	Prospective non-randomized Matched to historical control group	240	12 months	0–1 pad/24 h	RALP 77/79 (97) RRP 191/217 (88)
Son et al. (17)	2013	Prospective non-randomized	258	12 months	0–1 pad/24 h	RALP 146/146 (100) RRP /112 (98.2)
Kim et al. (18)	2018	Prospective non-randomized	529	12 months	0 pads/3 days and an absence of any unwanted urine leakage	RALP none or unilateral nerv-sparing 191/460 (41.5) RALP bilateral nerv-sparing 269/460 (58.5)
Olsson et al. (19)	2001	Prospective non-randomized	228	12 months	0 pads/24 h	LRP 29/37 (78.4)
Porpiglia et al. (13)	2012	Prospective randomized	120	12 months	0–1 pad/24 h	RALP 57/60 (95.0) LRP 50/60 (83.3)

*RALP, Robotic assisted laparoscopic radical prostatectomy; RRP, open retropubic radical prostatectomy; LRP, laparoscopic radical Prostatectomy*.

Importantly, the impact of urinary incontinence to affected patients is substantial and include stigmatization and significant reduction of quality of life (20). In addition, the cost burden of urinary incontinence is currently estimated between $19 and $32 billion in the USA (9).

Overactive bladder (OAB), with or without urinary incontinence, can also occur after radical prostatectomy and is an underestimated cause for urinary incontinence after radical prostatectomy. However, so far there is a lack of robust data for its incidence.

In this non-systematic review, we provide an overview on pathophysiology and current treatment options of male stress urinary incontinence after radical prostatectomy.

## Pathophysiology

There are different factors responsible for the post-rPR urinary incontinence. The most well-known factors include the changes that occur in the anatomy, the preoperative bladder function as well as the operation technique and the experience of the surgeon (21, 22). In addition, the anatomic support and the pelvic innervation have been identified as important contributors to post rPR continence (21). Among the many surgical and technical factors proposed in the literature as contributing to the development of urinary incontinence following rPR, extensive dissection during surgery, damage to the neurovascular bundle, and the development of postoperative fibrosis have a substantial negative impact on the continence status of men undergoing rPR. Sparing of the bladder neck and anterior, and possibly posterior, fixation of the bladder-urethra anastomosis are associated with better continence rates (22).

Continence is generally facilitated by the combination of the action of the detrusor muscle, the proximal intrinsic sphincter, the rhabdosphincter (23), and the urethral suspensory mechanism composed of pubourethral ligaments (24). After rPR, the proximal urethral sphincter, the suspensory ligaments as well as parts of the proximal intrinsic sphincter are removed. As a consequence, post rPR urinary continence is largely dependent on the rhabdosphincter (25). In addition, the pudendal nerve fibers that innervate the rhabdosphincter are damaged during the operation which has functional implications. This has been studied by transurethral ultrasound, that has shown thinning or atrophy as well as impaired contractility of the rhabdosphincter (25). Moreover, the innervation of the detrusor muscle and trigonum are impair which leads to a decreased detrusor contractility and poor bladder compliance (26, 27). The most predominant finding in urodynamic measurements is the sphincteric incontinence (28). On the other hand, intrinsic detrusor dysfunction and overactivity or impaired detrusor contractility, and altered detrusor compliance play a role in the post rPR continence (29). Preoperative urodynamic abnormalities have been observed to be present in 41% of patients, with half of them having detrusor overactivity (28).

About 50% of patients have preoperative impaired bladder compliance and impaired detrusor contractility and 47% *de novo* postoperative changes (30). Urodynamic studies carried out 1 year after rPR have shown sphincteric incontinence as the most common finding, which was responsible for about 88–100% urinary incontinence after rPR (26, 31, 32). About a third of the patients had an intrinsic sphincter deficiency as the single cause of their urinary incontinence (26, 32). Furthermore, detrusor overactivity and impaired bladder contractility were each found in up to a 30% of the cases (26, 32). However, in <9% of the cases, these findings were the only urodynamic finding (26, 32). In one out of five patients, bladder outlet obstruction was found, but this was the sole urodynamic finding in only 1% of the cases (31). Delayed first sensation (42%), mixed urgency-urge incontinence (48%), and decreased bladder capacity (< 300 mL) (41%) were the other findings on urodynamic measurements after rPR (26). It must also be stressed that, a highly well-established predictor of functional outcomes is the surgeon. It is well-known that patients treated in high volume centers and in experienced hands, are more likely to be dry. When reviewing different series, the absence of this variable could represent a limitation since, in some cases, an individual surgeon's outcomes may be much better, or worse, than any nomogram prediction. Better urinary continence recovery results can be expected by patients who undergo rPR performed by a surgeon with greater experience (33). An annual surgical case load of >50 cases/year results in improved continence recovery outcomes following rPR (33).

## OAB and Urgency Incontinence

In the context of management of post-rPR OAB syndrome, it is important to understand its underlying pathophysiological mechanism (34). Since OAB is multifactorial (35), the exact role of prostate surgery in the development of OAB is still under debate as, after rPR, there are several variables that could contribute to detrusor overactivity.

Detrusor overactivity in patients after radical prostatectomy has been mainly attributed to a partial denervation of the bladder during surgery (30). However, together with bladder denervation, other hypotheses, such as the urethrovesical mechanism, have been described.

It has been demonstrated that urethral afferents are activated by urethral perfusion (36) and they could modulate the micturition reflex via pudendal and pelvic afferent and efferent signals, causing bladder contraction. This has been described as urethrovesical mechanism (37–39).

In a recent study, Mastukawa et al. identified that low maximum urethral closure pressure at baseline and its decrease postoperatively were strong predictors of *de novo* post-rPR OAB underlying the role of the intrinsic sphincter deficiency on the pathophysiology of OAB (40).

In contrast, detrusor underactivity may cause OAB syndrome as well, which seems contradictory at the first glance. Bladder underactivity may affect up to 40% of patients after radical prostatectomy mostly due to denervation (41).

Bladder outlet obstruction is a known cause of OAB. The obstruction after RP is mainly caused by bladder neck contracture and urethral stricture due to the anastomosis of the bladder neck with the urethra, which has an incidence up to 20% (42). BOO causes damage to the smooth muscle demonstrating histological changes in the bladder wall causing spontaneous myogenic contractions (43). Therefore, the presence of infravesical obstruction due to urethral stricture or bladder neck contracture must be excluded.

## Predicting Urinary Incontinence After Radical Prostatectomy

Damage to the urethral sphincter complex, the surrounding structures, or their innervation leads to higher rates of urinary incontinence after rPR. In addition, certain biological factors and parameters known preoperatively, including older age, higher BMI, pre-existing LUTS, lower motor unit lesion, and functional bladder changes, have been identified to have a negative impact on continence rates after rPR (22).

**Figure 1:**
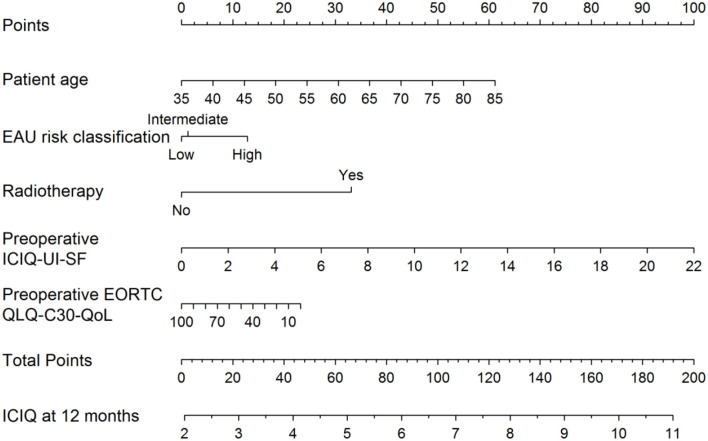
Nomogram for the preoperative prediction of the 12-month ICIQ-UI-SF score among patients diagnosed with prostate cancer and treated with robotic-assisted prostatectomy. Instructions: locate the patient's values for age, EAU risk classification, baseline EORCT QLQ-C30/QoL and baseline ICIQ-UI-SF on the corresponding axes. Draw a straight line up to the Points axis for each value to determine the number of points for that value. Calculate the sum of the values on the Points axis and locate this sum score on the Total Points axis. Draw a straight line down to find the patient's predicted ICIQ-UI-SF score at 12 months. From Tutolo et al. (44). EORCT QLQ-C30/QoL, European Organization for Research and Treatment for Cancer Quality of Life Questionnaire of Prostate Cancer; ICIQ-UI-SF, International Consultation on Incontinence Questionnaire-Urinary Incontinence Short Form; EAU, European Association of Urology.

**Figure 2:**
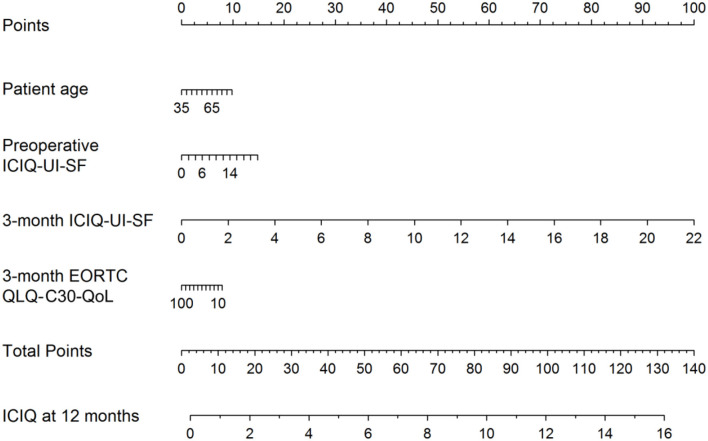
Nomogram for the postoperative prediction of the 12-month ICIQ-UI-SF score among patients diagnosed with prostate cancer and treated with robotic-assisted laparoscopic prostatectomy. Instructions: locate the patient's values for age, 3-month EORCT QLQ-C30/QoL, intraoperative complications, preoperative ICIQ-UI-SF and 3-month ICIQ-UI-SF on the corresponding axes. Draw a straight line up to the Points axis for each value to determine the number of points for that value. Calculate the sum of the values on the Points axis and locate this sum score on the Total Points axis. Draw a straight line down to find the patient's predicted ICIQ-UI-SF score at 12 months. Taken From Tutolo et al. (44). EORCT QLQ-C30/QoL, European Organization for Research and Treatment for Cancer Quality of Life Questionnaire of Prostate Cancer; ICIQ-UI-SF, International Consultation on Incontinence Questionnaire-Urinary Incontinence Short Form.

Recently, a preoperative model was presented to predict incontinence before rPR (Figure 1) (44). According this nomogram, high risk for biochemical recurrence, adjuvant radiotherapy, lower results in the validated quality of life questionnaire EORCT QLQ-C30/QoL, higher sum score of the validated questionnaire International Consultation of Incontinence Questionnaire—Urinary Incontinence—Short form (ICIQ-UI-SF) and higher patient age, were associated with statistically significant higher sum scores of the 12-month ICIQ-UI-SF, thus, representing higher impact of urinary incontinence (Figure 1) (44). Together with the preoperative model a new, postoperative nomogram was introduced to inform patients about the probability of an additional surgery for incontinence or, on the other hand, about the importance of enduring with a strict pelvic floor muscle training protocol (Figure 2) (44).

Interestingly, these results did not show any association with ICIQ-UI-SF, when including surgery volume (namely <50, 50–100, or >100 cases per year) (44).

R-squared (*R*^2^), the statistical measure that represents the proportion of the variance for a dependent variable, equalled 4% and 43% in the preoperative and postoperative models, respectively. This is mainly due to the retrospective nature of the study and to the intrinsic characteristics of the database (strict rules of the Belgian Cancer registry). The major drawback of this study, together with its retrospective nature, is that a single dataset has been used for development and validation of the model. Although a non-random splitting of the data is an acceptable design for evaluating model performance, external validation still has to be performed (42).

## Treatment of Male Stress Urinary Incontinence

### Conservative and Pharmacologic Therapy

Although there is conflicting evidence regarding the importance of conservative treatment after post-prostatectomy urinary incontinence (45), pelvic floor muscle training (PFMT) is still considered as the first treatment choice (46). Duloxetin, a serotonin/norepinephrine reuptake inhibitor, either alone or in combination with PFMT, may hasten recovery of urinary incontinence but is often associated with severe gastrointestinal and central nervous side effects (47, 48). However, neither PFMT nor duloxetine may cure male stress urinary incontinence.

### Surgical Therapy

If conservative therapy fails, surgical treatment options should be offered to the patients. The artificial urinary sphincter (AUS) has been considered the gold standard for several decades. In a recent study urinary incontinence rates remained high, with no evidence of difference between male sling and AUS (49). The mode of function of AUS is a circumferential compression of the urethra based on a hydraulic mechanism. Nowadays, several alternative procedures with different operating principles compete against the AUS. These procedures are classified to bulking agents, male slings, and compressive devices. Table 2 presents success and complications rates of different treatment options of selected clinical trials and Figure 2 demonstrates the different surgical devices *in situ*. Table 2 presents success and complications rates of different treatment options of selected clinical trials and Figure 2 demonstrates the different surgical devices *in situ*.

**Table 2:** Continence and complications rates after different treatment modalities of male stress urinary incontinence in selected clinical trials.

**References**	**Device**	**Device type**	**Year**	**Study design**	**Number of patients**	**Follow-up time**	**Continence definition**	**Urinary continence, n/N (%)**	**Complications**
Bauer et al. (50)	AdVanceXP	Fixed sling	2015	Prospective non-randomized	94	24 months	0 pads and 0–5 g in 24 h pad test	35/46 (73.1)	Urinary tract infection *n* = 1, wound infection *n* = 2, urgency *n* = 3, explantation due to pain *n* = 2 or ineffectiveness *n* = 3
Collado Serra et al. (51)	AdVance AdVanceXP	Fixed sling	2013	Prospective non-randomized	61	26 months	0 pads/24h	49/61 (80.0)	Acute urinary retention *n* = 9(15%), perineal scrotal pain *n* = 5(8%), perineal hematoma *n* = 2(3%), *deNovo* urgency *n* = 5(8%)
Friedl et al. (52)	ATOMS	Adjustable sling	2017	Retrospective non-randomized	287	31 months	0–1 pad/24 h, <10 ml/day	184/287 (64.0)	Urinary retention *n* = 8(3%), early infection *n* = 6 (2%), hematoma *n* = 6(2%), removals *n* = 56 (20%) due to titanium intolerance *n* = 23 (41%), leak *n* = 12(21%), early infection *n* = 6 (11%) late infection *n* = 6(11%), dysfunction *n* = 5(9%), dislocation *n* = 3(5%), persistent pain *n* = 1(2%) reimplantation *n* = 29 (52%), solitary port change *n* = 14(5%), AUS solution *n* = 11 (4%)
Mühlstädt et al. (53)	ATOMS	Adjustable sling	2016	Retrospective nonrandomised	54	27.5 months	0–1 pad/24 h	26/54 (48.1)	Scrotal hematoma *n* = 2 (3.7%), pain *n* = 3 (5.6%), urinary retention *n* = 1 (1.9%), woundinfection perineal *n* = 2 (3.7%), wound infection port- *n* = 4 (7.4%), port erosion *n* = 1 (1.9%), incipient erosion of the port *n* = 2 (3.7%)
Cornel et al. (54)	Argus	Adjustable sling	2016	Prospective non-randomized	36	12 months	0–1 pad/24 h 0–2 g/24 h	29/36 (82.9)	Urinary retention *n* = 7, Hematoma *n* = 1, insensibility scrotum *n* = 4, perineal pain <6 months *n* = 9, urinary tract infection *n* = 1, wound infection *n* = 6, inguinal wound reclosure removal sling column *n* = 3, sling infection and removal *n* = 3
Lima et al. (55)	Argus AdVance	Adjustable sling Fixed sling	2016	Prospective non-randomized	44	36.2 months 33.1 months	0–1 pad/24 h	23/25 (92) 16/19 (84)	Argus: *n* = 1 (4%) urinary retention, Revision surgery for incontinence *n* = 6 (24%) AdVance: *n* = 2 (11%)urinary retention
Leizour et al. (56)	Remeex	Adjustable sling	2017	Prospective non-randomized	25	31 months	0 pad/24 h	9/25 (41)	Explantation *n* = 1, infection *n* = 3.
Rocha et al. (57)	AMS800	Artificial urinary sphincter	2008	Prospective non-randomized	40	53 months	0 pad/24 h	20/40 (50.0)	Perineal hematoma *n* = 1, device infection *n* = 3 (7.5%), mechanical failure *n* = 2(5%), urethral atrophy *n* = 2(5%), overactive bladder syndrome *n* = 4 (10%)
Kaiho et al. (58)	AMS800	Artificial urinary sphincter	2018	Prospective non-randomized	135	12 months	0 pads	27/93 (37.3)	Wound infection *n* = 5, urinary retention *n* = 4 hematoma *n* = 2, others *n* = 2, mechanical failure *n* = 7, late infection *n* = 4, urethral erosion *n* = 3, urethral erosion and infection *n* = 1, pump malposition *n* = 1,
Yiou et al. (59)	ProAct	Non-circumferential compressive	2015	Prospective non-randomized	20	12 months	0 pads	12/18 (66.7)	Late infection of perineal wound *n* = 2 due to additional InVance implantation
Crivellaro et al. (60)	ProAct	Non-circumferential compressive	2008	Prospective non-randomized	46	19 months	0–1 pad/24 h	30/44 (68.0)	Erosion *n* = 2, spontaneous deflation *n* = 1, infection *n* = 1, migration *n* = 2, explantation *n* = 6

### Bulking Agents

Theoretically, bulking agents may be an attractive treatment option for patients with limited amount of urine loss, unfit for surgery, or unwilling for surgery with implantable devices (61). However, bulking agents have been discredited due to various complications, such as embolization, migration, absorptions, allergic, and fibrotic reactions. Novel bulking agents are characterized by their non-migrating and non-absorbable properties (62). Although bulking agents are commonly used in female SUI, evidence regarding the treatment of male SUI is scarce. Moreover, there is no standardized surgical technique regarding amount and position of injection. In a recent systematic review of bulking agents utilized for male SUI including polydimethylsiloxane elastomer (Macroplastique), polyacrylate polyalcohol copolymer (Opsys), carbon coated zirconium (Durasphere), and vinyl dimethyl terminated poly-dimethylsiloxane polymer (Urolastic), no final conclusion could be drawn due to the high risk of bias, incoherent reporting of urinary incontinence and surgical technique and contradictory results (61). It can be concluded that, there is currently, no recommendation for the utilization of bulking agents for the treatment of male stress urinary incontinence outside of clinical trials (46).

### Male Slings

Male slings are minimally invasive procedures where a sling is positioned under the bulbar urethra through a retropubic or transobturator approach (46). They are distinguished into fixed and adjustable slings.

### Fixed Slings

The mode of function of fixed slings is the reposition of the urethra to a proximal position without affecting the sphincter mechanism directly. This mechanism bases on the hypothesis, that urinary incontinence with residual sphincter function is caused by a urethral or perineal descent which is associated with lacity, iatrogenic causes, or aging in the levator ani complex (63). The distal urethral sphincter may be supported indirectly by a hammock underneath the urethral bulb though increasing the coaptative zone within the sphincteric membranous urethra. During increased physical exercise the blood flow is accumulated within the supported corpus spongiosum and hereby increases the zone of coaptation which is enabled by the male sling (7).

However, the current considerations base on the existence of an at least partial or complete presence of the urethral sphincter. Therefore, fixed slings are indicated in patients with mild to moderate male SUI (46) whereas, higher degrees of urinary incontinence should be reserved to compressive devices.

The most investigated fixed male sling is the AdVance, and second generation AdVanceXP (Boston Scientific, Marlborough, Massachusetts, USA). In mid-term follow up of the AdVanceXP in a selected patient population, 68.8% and 22.8% of the patients were either cured or improved, respectively, with a mean urine loss decreased to 19.1 g. Importantly, no intraoperative or long-term complications occurred in either of these patients (64). In a recent meta-analysis, the objective cure rates for fixed slings were reported between 8.3 and 87%. Pain was the most common complication although chronic pain was only reported in 1.3%. The second most common complication is urinary retention but being mostly a temporary condition (65).

### Adjustable Slings

**Figure 3:**
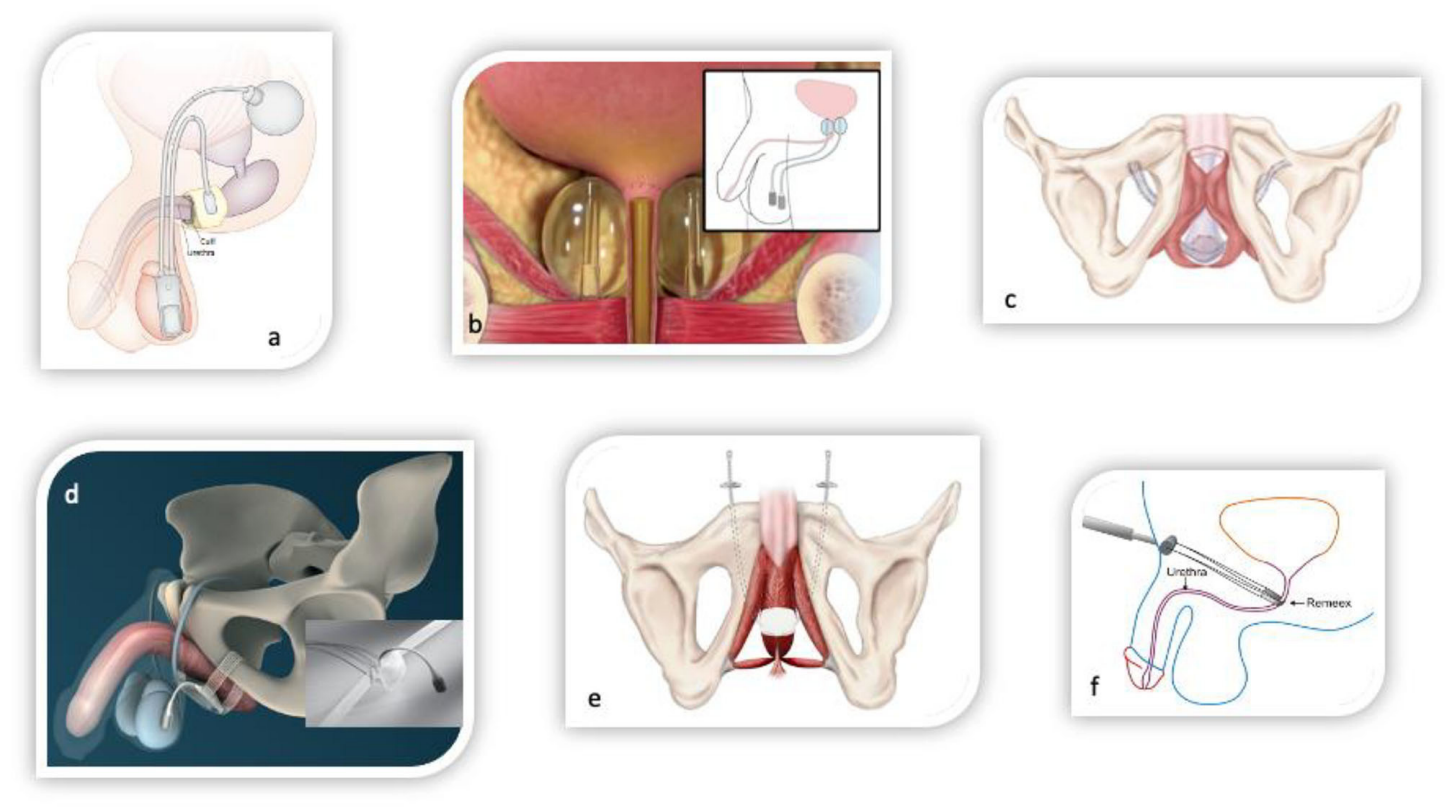
Surgical devices for the treatment of male stress urinary incontinence. **(a)** Circumferential compressive three-piece artificial urinary sphincter AMS800 (Boston scientific, USA). **(b)** Non-circumferential compressive device ProACT (UroMedica, USA). **(c)** Fixed male sling AdVanceXP (Boston Scientific, USA). **(d)** Adjustable male sling ATOMS (A.M.I., Austria). **(e)** Adjustable male sling Argus (Promedon, Argentina). **(f)** Adjustable male sling Remeex (Neomedic, Spain).

Adjustable slings offer the possibility of adjuvant adaptation of the sling tension or compression of the urethra by either tighten the sling arms or filling a cushion, which is localized beneath the urethra. The mode of function of adjustable slings are therefore complemented by the possibility of mechanical compression of the urethra (Figure 3).

Currently, there are three commercialized adjustable sling available: Remeex (Neomedic, Madrid, Spain), Argus (Promedon, Cordoba, Argentina), and ATOMS (A.M.I., Feldkirch, Austria). The currently most investigated adjustable sling is the ATOMS. In a recent meta-analysis including a total of 1.393 patients with an ATOMS, the mean cure rate was 67% and improvement of urinary SUI was 90%. The complication rate was 16.5% although major complications occurred in only 3% (66). Including all adjustable slings, the cure rate is reported between 17 and 92% in a meta-analysis. Chronic painful condition was 1.5% and the most common complication is infection with subsequent explantation of the device (65). These results are accordance with a large cohort trial, reporting a significant higher infection rate of 2.3% and pain rate of 11.9%. The total explantation rate was 4.0% (67). Furthermore, it could be demonstrated that adjustable slings are more commonly utilized in patients with higher degree of SUI and risk factors, although functional outcomes remained comparable to fixed slings.

In conclusion, there might be beneficial cure rates in adjustable slings in comparison to fixed slings. However, complications rates might be higher in adjustable slings.

### Compressive Devices

Compressive devices can be distinguished between circumferential and non-circumferential devices.

### Circumferential Compressive Devices

The AUS is a three-piece device including an urethral cuff, pump, and reservoir. The mode of function is a mechanical circumferential compression of the urethra and is based on a hydraulic mechanism. The most investigated device is the AMS800 (Boston Scientific, Marlborough, Massachusetts, USA). Its predecessor has been introduced in 1972 (68) and is available in the current shape since 1982 (69). The continence rate of the AUS are reported between 61 and 100% (70) and in a long-term analysis with a mean of 15 years, the continence rate was still 77.2%. Including any degree of urinary incontinence independently of the existence of the urethral sphincter. Therefore, the AUS is recommended for the treatment of moderate to severe male SUI and in particular in patients with a history of pelvic irradiation or urethral stricture disease.

Despite the favorable functional outcome, the AUS is associated with higher complications rates than male slings (71). The mean rate of infection and erosion in a pooled analysis was 8.5%, mechanical failure 6.2%, urethral atrophy 7.9%, and the mean rate of reintervention was 26%. Nevertheless, in particular patients with higher degree of urinary incontinence facing limited treatment options. If the AUS fails, the ultima ratio is urinary diversion.

Apart from the AMS800, which offer the largest amount of literature and follow-up time, there are several other commercialized AUS available. Victo (Promedon, Cordoba, Argentina) is three-piece device similar to the AMS800 but offers additional the possibility to adjust the device by increasing the intraluminal pressure through percutaneous fluid injection into a port which is located in the bottom of the pump. Zephyr (Zephyr Surgical Implants, Geneva, Switzerland) offers a two-piece device including only a pump and an urethral cuff. Furthermore, the device also offers the possibility of postoperative adjustability in a similar approach as described.

### Non-circumferential Compressive Devices

ProAct (Uromedica, Plymouth, USA) is a non-circumferential compressive device. The mechanism is based on two balloons which are positioned lateral to the proximal urethra. The balloons are filled in an ambulatory matter and results in a mechanical compression of the urethra. The success rates are reported between 62 and 68% accompanied by explantation rate of 12.3%. The most common complications are erosion (3.2–10.9 %) and dislocation (4–6.2 %) (72). Other prospective series even reported complication rates between 11 and 58% (46). There is currently no direct recommendation for the utilization of ProAct in mal SUI in the European guidelines. However, in the summary of very limited evidence, it is evaluated as effective in short term, although associated with a high risk of complications and should not be offered to patients with a history of pelvic irradiation (46).

## Future Perspectives and Conclusions

Prostate cancer is one of the most problematic and frequently encountered malignancies in male patients. It often occurs when men are still in the active period of their lives. Consequently, there is a high demand for minimally invasive therapeutic approaches, susceptible of preserving urinary continence and sexual function. Unfortunately, stress urinary incontinence is a common adverse event in men with localized or locally advanced prostate cancer undergoing radical prostatectomy, but also secondary to radiotherapy (external beam radiotherapy as well as brachytherapy) and to cryosurgery (73).

Despite rehabilitative procedures such as pelvic floor muscle training, biofeedback, electrical stimulation, lifestyle changes, or a combination of these strategies, no fully efficient treatment alternative has yet been established for this pathology (74). On the other hand, it should be acknowledged that nursing care, including the understanding of the patient's needs, education, and psychosocial support remain essential features while aiming to improve the quality of life of prostate cancer patients.

Concerning the newest experimental treatments made available for urinary incontinence subsequent to prostate cancer surgery, there are studies that have shown a significant improvement of continence after ultrasound guided injection of fibroblasts and myoblasts into the sphincter (75). Other clinical trials also emphasized encouraging outcomes provided by stem-cells injection into the rhabdosphincter (76). Last but not least, promising outcomes have been outlined as a result of intravesical Onabotulinum toxin A injection (77).

Most importantly, the therapeutic decision and the chosen treatment option must be individualized for each patient according to clinical and social factors. During the recent years, the development of new therapeutic choices such as male sling techniques provided a more acceptable management pathway for less severe forms of urinary incontinence related to radical prostatectomy. Following this perspective, technological improvements and the emergence of new dedicated devices currently create the premises for a continuously positive evolution of clinical outcomes in this particular category of patients.

## Author Contributions

MR: data collection, coordination and drafting the manuscript, and supervising. TM and TH: data collection, drafting the manuscript, and supervising. BG and MT: data collection and drafting the manuscript.

## Conflict of Interest

The authors declare that the research was conducted in the absence of any commercial or financial relationships that could be construed as a potential conflict of interest.
